# Malay Version of the mHealth App Usability Questionnaire (M-MAUQ): Translation, Adaptation, and Validation Study

**DOI:** 10.2196/24457

**Published:** 2021-02-04

**Authors:** Norashikin Mustafa, Nik Shanita Safii, Aida Jaffar, Nor Samsiah Sani, Mohd Izham Mohamad, Abdul Hadi Abd Rahman, Sherina Mohd Sidik

**Affiliations:** 1 Dietetics Program and Center for Community Health Study Faculty of Health Sciences Universiti Kebangsaan Malaysia Kuala Lumpur Malaysia; 2 Department of Nutrition Science Kulliyyah of Allied Health Sciences International Islamic University Malaysia Kuantan, Pahang Malaysia; 3 Department of Psychiatry Faculty of Medicine and Health Sciences Universiti Putra Malaysia Serdang, Selangor Malaysia; 4 Primary Care Unit Faculty of Medicine and Defence Health Universiti Pertahanan Nasional Malaysia, Malaysia Sg Besi, Wilayah Persekutuan Kuala Lumpur Malaysia; 5 Center for Artificial Intelligence Technology Faculty of Information Sciences and Technology Universiti Kebangsaan Malaysia Bangi, Selangor Malaysia; 6 Sports Nutrition Center National Sport Institute Bukit Jalil, Kuala Lumpur Malaysia

**Keywords:** mHealth app, questionnaire validation, questionnaire translation, Malay MAUQ, usability, mHealth, education, usability, Malay language, Malay, questionnaire, mobile phone

## Abstract

**Background:**

Mobile health (mHealth) apps play an important role in delivering education, providing advice on treatment, and monitoring patients’ health. Good usability of mHealth apps is essential to achieve the objectives of mHealth apps efficiently. To date, there are questionnaires available to assess the general system usability but not explicitly tailored to precisely assess the usability of mHealth apps. Hence, the mHealth App Usability Questionnaire (MAUQ) was developed with 4 versions according to the type of app (interactive or standalone) and according to the target user (patient or provider). Standalone MAUQ for patients comprises 3 subscales, which are ease of use, interface and satisfaction, and usefulness.

**Objective:**

This study aimed to translate and validate the English version of MAUQ (standalone for patients) into a Malay version of MAUQ (M-MAUQ) for mHealth app research and usage in future in Malaysia.

**Methods:**

Forward and backward translation and harmonization of M-MAUQ were conducted by Malay native speakers who also spoke English as their second language. The process began with a forward translation by 2 independent translators followed by harmonization to produce an initial translated version of M-MAUQ. Next, the forward translation was continued by another 2 translators who had never seen the original MAUQ. Lastly, harmonization was conducted among the committee members to resolve any ambiguity and inconsistency in the words and sentences of the items derived with the prefinal adapted questionnaire. Subsequently, content and face validations were performed with 10 experts and 10 target users, respectively. Modified kappa statistic was used to determine the interrater agreement among the raters. The reliability of the M-MAUQ was assessed by 51 healthy young adult mobile phone users. Participants needed to install the MyFitnessPal app and use it for 2 days for familiarization before completing the designated task and answer the M-MAUQ. The MyFitnessPal app was selected because it is one among the most popular installed mHealth apps globally available for iPhone and Android users and represents a standalone mHealth app.

**Results:**

The content validity index for the relevancy and clarity of M-MAUQ were determined to be 0.983 and 0.944, respectively, which indicated good relevancy and clarity. The face validity index for understandability was 0.961, which indicated that users understood the M-MAUQ. The kappa statistic for every item in M-MAUQ indicated excellent agreement between the raters (κ ranging from 0.76 to 1.09). The Cronbach α for 18 items was .946, which also indicated good reliability in assessing the usability of the mHealth app.

**Conclusions:**

The M-MAUQ fulfilled the validation criteria as it revealed good reliability and validity similar to the original version. M-MAUQ can be used to assess the usability of mHealth apps in Malay in the future.

## Introduction

Mobile health (mHealth) plays a vital role in delivering health education and disease management advice and in monitoring patients’ health in many ways [1,2]. mHealth development is beneficial not only for populations with diseases but also for active individuals, athletes, and older adults [3-6]. The 2 main factors that influence the effective use of mHealth apps for self-care are perceived usefulness and ease of use [7]. Well-designed mHealth apps have proved their cost-effectiveness by educating and empowering patients as well as by improving the medication adherence of patients [8-10]. However, reviews have shown that there is insufficient evidence for the quality of mHealth apps.

The target users of mHealth apps can be categorized into either patients or health care providers. mHealth apps for patients are specifically designed for those who intend to manage their health, for example, health behaviors, while mHealth apps for health care providers are developed for delivering health care services, for example, medication prescription, laboratory orders, consultation, and patient education [11]. Some terms need to be customizable in the usability questionnaire to represent specific target users of mHealth devices. In addition to categorization according to target users, mHealth apps can be categorized according to interactive functions, that is, interactive mHealth app or standalone mHealth app [11]. Interactive mHealth apps enable users to send and receive information from their health care providers. Patient-provider communication can be synchronous or asynchronous. Standalone mHealth apps contain reminders or progress charts that collect health or activity information; however, the data are not shared with the health care providers immediately. Therefore, there are limited interactions in standalone mHealth apps [11].

Any mobile app should be designed with good usability or the app should be easy to use without errors and be able to achieve its objectives effectively. The characteristics of good usability are (1) efficiency: the comprehensiveness of a product enables users to achieve their goals, (2) satisfaction: users have positive feedback about the product, (3) learnability: the product is easy to learn, (4) memorability: the usage mechanism of the product or the system is easy to remember even after the users have not been using it for a while, and (5) low error rate: low errors can prevent disasters [12,13]. However, the above usability characteristics are not specific to mHealth apps because of several phone issues, for example, small screen or screen overload [14].

A new usability model has been proposed to enhance the framework within the context of mobile apps and it is called PACMAD (People At the Centre of Mobile App Development) [15]. This model identifies 3 factors that should be considered when evaluating usability: (1) user, (2) task, and (3) context of use that can affect the overall usability of a mobile app [15]. The added element of “cognitive load” in PACMAD assesses the user ability to perform additional tasks, for example, exercise while using the mobile device [15]. Therefore, usability should be based on effectiveness, efficiency, satisfaction, learnability, memorability, and cognitive load with very minimal errors [15].

Several usability scales have been evaluated based on the importance of usability in developing mobile apps. The mobile app rating scale and the user version of the mobile app rating scale include some usability components to assess the quality of mobile apps [16,17]. However, they does not assess the usability by the end user and they have a broader scope [16,17]. Taking this into consideration, mobile apps that are intended for health purposes (mHealth apps) should be assessed with a more appropriate usability questionnaire. Therefore, a new usability scale called the mHealth App Usability Questionnaire (MAUQ) was developed [18]. This MAUQ can assess the ease of use, interface, satisfaction, and the usefulness of mHealth apps to the end users (either patients or health care providers) and the type of interaction between the patients and health care providers (standalone or interactive).

To the best of our knowledge, only 1 study has reported the usability of a Malay-translated mobile app [19]. However, the questionnaire used was the system usability scale questionnaire, which caters to general software systems. As more health interventions using mobile apps are being developed, this study aims to translate and validate a Malay version of MAUQ for future mHealth app research and usage.

## Methods

### Overview of the Questionnaire

The English version of MAUQ was developed and validated by Zhou and his team [11]. The aim of MAUQ was to assess the usability of mHealth apps among patients and health care providers. MAUQ has 4 versions assessing the type of app (interactive or standalone) and the target users of the app (patient or health care provider). For this study, “MAUQ standalone used by the patient” was used. The patient refers to a person who uses an mHealth app to maintain, improve, or manage his/her health. The questionnaire consists of 3 subscales, which are ease of use (5 items), interface and satisfaction (7 items), and usefulness (6 items). Participants rate each of the items using a 7-point Likert scale ranging from 1 (strongly disagree) to 7 (strongly agree). The usability of the app is determined by the total and average of all statements—the higher the overall average, the better the usability of the app. However, if the average score is lower than 4, it means that the usability of the app is not good. MAUQ standalone used by patients had strong internal consistency with an overall Cronbach α value of .914 [18]. This study was conducted from April to August 2020.

### Translation and Adaptation Process

The original MAUQ standalone questionnaire was translated into Malay using the guidelines for health care translation and cross-cultural adaptation to achieve equivalence between the original and the translated version [20]. First, the forward translation process (from English to Malay) was conducted by 2 independent certified translators who knew the Malaysian culture and linguistics; 1 translator was specialized in the computer science field while the other was specialized in health sciences. Thus, 2 independent documents (F1 and F2) were produced. Second, the documents (F1 and F2) underwent harmonization. In this process, a third translator was appointed (in this step, we used a Malay language teacher with teaching experience of more than 5 years) to identify any ambiguities and discrepancies in the words, sentences, grammar, and meaning by comparing between F1 and F2 and between the original MAUQ and both F1 and F2. The discrepancies and ambiguities were discussed and resolved through consensus decision making by the third translator, the first 2 translators, and 2 members of the research team (NM and AJ) to produce an initial translated version of the MAUQ document (H1). Third, the translated version of the MAUQ (H1) was given to 2 independent native Malay speakers who spoke English as their second language. One was the content expert of the build instrument (computer science lecturer) while the other one did not know the instrument construct (English language teacher). Both had never seen the original version of MAUQ. They produced 2 independent documents of the back-translated version of MAUQ (B1 and B2). Fourth, a committee was approached to compare the documents with the original MAUQ. This committee comprised of 2 researchers (NM and AJ), translators in the first and third steps, and the original developer of MAUQ. The discussion among the committee members was conducted through emails and web-based meetings using the Google meet platform. Any ambiguity and inconsistency in the cultural meaning and expression of the words and sentences of the items and answer format were addressed and resolved through consensus among committee members to derive the prefinal adapted questionnaire. This adapted questionnaire was called the Malay version of the mHealth Usability Questionnaire (M-MAUQ).

### Validation of the Questionnaire

The M-MAUQ underwent a process of validation that consisted of content validity, face validity, and reliability (internal consistency). Content validation aimed to evaluate the relevancy of the items in each domain and the clarity of the translated item to assess the usability of mobile apps related to health. The 10 experts who conducted content validation of the M-MAUQ were 4 mobile app developers, 3 PhD senior lecturers in computer science and information technology, and 3 health-related practitioners. They were asked to give a score of 1 (item not relevant/clear) to 4 (item very relevant/clear). The establishment of content validity was represented by the content validity index value in the form of item-level content validity index and overall-scale content validity index. Before content validity index was calculated, scores of 3 and 4 were recategorized as 1 (relevant/clear) and scores of 1 and 2 as 0 (not relevant /not clear). Next, the number of experts in agreement (relevant/clear) was divided by the total number of experts. The items that had content validity index of at least 0.79 indicated that the item was relevant to the domain, clear, and comprehensible to the target users [21].

Face validation testing, which aims to assess the clarity and comprehensibility of the translated items, was conducted by 10 target users. The users were asked to give scores from 1 (item not understandable) to 4 (item very understandable) based on the understandability of the translated items in M-MAUQ. Scores of 3 and 4 were recategorized as 1 (understandable) and scores of 1 and 2 as 0 (not understandable). The face validity index was computed by calculating the scale average. They highlighted the words that they could not understand. The interrater agreement among the 10 experts and the 10 target users was determined using the modified kappa statistic. The probability of chance agreement (Pc) was first determined for each item using the following formula:


Pc = [(N!/A!) (N–A)!] * 0.5^N^

In this formula, N is the number of raters (experts/target user) and A is the number of experts/target users who agree that the item was clear or relevant or understandable [21]. Next, kappa was determined using the following formula:


κ = (item-level content validity index–Pc) / (1–Pc)

The kappa formula was computed using Microsoft Excel, with a value above 0.74 considered as excellent, 0.60 to 0.74 as good, and 0.54 to 0.59 as fair [21]. For reliability testing, a sample size of 38 participants was calculated, with an expected Cronbach α of .80 and an expected precision of 0.1 at 95% CI [22]. With an expected dropout rate of 20% taken into account, the final sample size calculated was 48 [22].

Participants were recruited among students in the International Islamic University of Malaysia, Kuantan Campus through invitation using the WhatsApp group by student representatives. The student representative contact was obtained with his/her permission from the department administrative office. Participants interested in joining the study were given a link to a new WhatsApp group where further instructions were provided. A web-based consent form and the flow of the study were shared to the WhatsApp group. Participants who agreed to join had to complete the web-based informed consent form. The inclusion criteria of the participants were (1) no known medical illness and (2) owned a smartphone. They were asked to install the MyFitnessPal app on their mobile phone and use it multiple times for 2 days before the session in order to be familiar with the features. The MyFitnessPal app was selected for the usability study because it is among the most popular installed mHealth apps globally; it has been used in most studies and is available for Android and iPhone users [23,24]. Moreover, the MyFitnessPal app is considered a standalone mHealth app.

A general introduction and a brief demo of the MyFitnessPal app was provided on the Google meet platform. When using the MyFitnessPal app, participants were asked to finish the following tasks: (1) create own profile and determine their weight goals, (2) identify their remaining calories for that day, which included calorie goals, calories in (food), and calories out (exercise), (3) use diary features to add new records about food eaten and water consumed on that day and view the total nutrient intake (macronutrient and micronutrient), and (4) explore health-related and nutrition-related articles, recipes, and plan features. After finishing the tasks, participants were asked to answer the M-MAUQ. Web-based data were collected due to the current COVID-19 pandemic, because mass gathering was not recommended. The internal consistency of M-MAUQ was evaluated by calculating the Cronbach α value for the entire questionnaire and its subscale. Higher α value suggests greater internal reliability and more than .70 is acceptable as good internal reliability [25].

**Figure 1:**
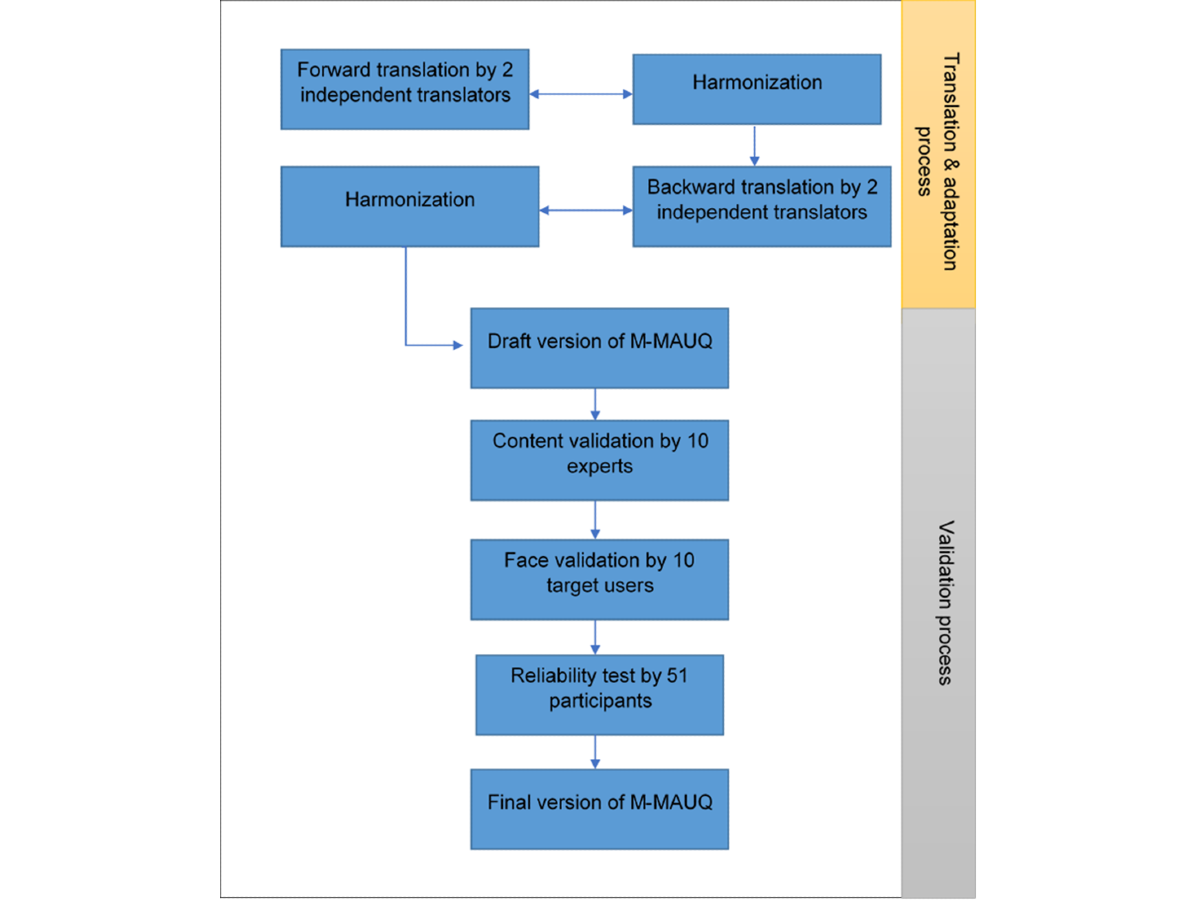
The flow of translation, cross-cultural adaptation, and validation process for the Malay version of the mHealth App Usability Questionnaire (M-MAUQ).

The validation tests performed on M-MAUQ were conducted using a web-based Google form, where the link was sent to each participant via personal WhatsApp (for the content and face validity test) or group WhatsApp (for the reliability test) to facilitate data collection. Figure 1 illustrates the overview of the flow of the translation and adaptation process, together with the validity process. All statistical analyses were performed using SPSS statistics version 23 (IBM Corp). This study obtained ethical approval from the UKM Research Ethics Committee (Ref No. UKM PPI/111/8/ JEP-2019-008) and was funded by the National Sports Institute of Malaysia (Grant code: NN-2018-093).

## Results

The content validity index for the relevancy (Table 1) and clarity (Table 2) of M-MAUQ was calculated to be 0.983 and 0.944, respectively. Meanwhile, the face validity index (Table 3) for understandability was calculated to be 0.961. The content validity index and face validity index scores above 0.79 indicate that the items in the questionnaire are relevant for domain, clarity, and understandability for the target user [26]. The modified kappa agreement for every item in M-MAUQ demonstrated excellent agreement (κ=0.76-1.09).

**Table 1:** Content validity index of item relevancy and modified kappa agreement by 10 experts.

Items	Rating of 3 or 4 (n)	Rating of 1 or 2 (n)	Item-level content validity index^a^	Probability of chance occurrence^b^	Modified kappa^c^	Interpretation
1	10	0	1.000	0.001	1.000	Excellent
2	10	0	1.000	0.001	1.000	Excellent
3	10	0	1.000	0.001	1.000	Excellent
4	9	1	0.900	0.010	0.899	Excellent
5	10	0	1.000	0.001	1.000	Excellent
6	10	0	1.000	0.001	1.000	Excellent
7	10	0	1.000	0.001	1.000	Excellent
8	10	0	1.000	0.001	1.000	Excellent
9	10	0	1.000	0.001	1.000	Excellent
10	9	1	0.900	0.010	0.899	Excellent
11	10	0	1.000	0.001	1.000	Excellent
12	10	0	1.000	0.001	1.000	Excellent
13	10	0	1.000	0.001	1.000	Excellent
14	10	0	1.000	0.001	1.000	Excellent
15	10	0	1.000	0.001	1.000	Excellent
16	10	0	1.000	0.001	1.000	Excellent
17	9	1	0.900	0.010	0.899	Excellent
18	10	0	1.000	0.001	1.000	Excellent

^a^Overall-scale content validity index=0.983.

^b^Computed using the formula: Pc = [(N!/A!) (N–A)!] * 0.5^N^, where Pc=probability of chance occurrence, N=number of experts, and A=number of experts who agree the items are relevant.

^c^Computed using the formula: κ = (item-level content validity index – Pc) / (1–Pc), where Pc=probability of chance occurrence. The interpretation criteria for kappa were as follows: Excellent=κ>0.7, Good=0.6≤κ≤0.74, and Fair=0.40≤κ≤0.59.

**Table 2:** Content validity index of item clarity and modified kappa agreement by 10 experts.

Items	Rating of 3 or 4 (n)	Rating of 1 or 2 (n)	Item-level content validity index^a^	Probability of chance occurrence^b^	Modified kappa^c^	Interpretation
1	10	0	1.000	0.001	1.000	Excellent
2	10	0	1.000	0.001	1.000	Excellent
3	8	2	0.800	0.176	0.757	Excellent
4	10	0	1.000	0.001	1.000	Excellent
5	10	0	1.000	0.001	1.000	Excellent
6	9	1	0.900	0.010	0.899	Excellent
7	10	0	1.000	0.001	1.000	Excellent
8	8	2	0.800	0.176	0.757	Excellent
9	7	3	0.700	4.219	1.093	Excellent
10	9	1	0.900	0.010	0.899	Excellent
11	10	0	1.000	0.001	1.000	Excellent
12	10	0	1.000	0.001	1.000	Excellent
13	10	0	1.000	0.001	1.000	Excellent
14	10	0	1.000	0.001	1.000	Excellent
15	10	0	1.000	0.001	1.000	Excellent
16	10	0	1.000	0.001	1.000	Excellent
17	10	0	1.000	0.001	1.000	Excellent
18	9	1	0.900	0.010	0.899	Excellent

^a^Overall-scale content validity index=0.944.

^b^Computed using the formula: Pc = [(N!/A!) (N–A)!] * 0.5^N^, where Pc=probability of chance occurrence, N=number of experts, and A=number of experts who agree the items are relevant.

^c^Computed using the formula: κ = (item-level content validity index – Pc) / (1–Pc), where Pc=probability of chance occurrence. The interpretation criteria for kappa were as follows: Excellent=κ>0.7, Good=0.6≤κ≤0.74, and Fair=0.40≤κ≤0.59.

**Table 3:** Face validity index of item understandability and modified kappa agreement by 10 target users.

Items	Understand (rating of 3 or 4) (n)	Not understand (rating of 1 or 2) (n)	Item-level face validity index^a^	Probability of chance occurrence^b^	Modified kappa^c^	Interpretation
1	10	0	1.000	0.001	1.000	Excellent
2	10	0	1.000	0.001	1.000	Excellent
3	8	2	0.800	0.176	0.757	Excellent
4	9	1	0.900	0.010	0.899	Excellent
5	10	0	1.000	0.001	1.000	Excellent
6	8	2	0.800	0.176	0.757	Excellent
7	10	0	1.000	0.001	1.000	Excellent
8	10	0	1.000	0.001	1.000	Excellent
9	10	0	1.000	0.001	1.000	Excellent
10	8	2	0.800	0.176	0.757	Excellent
11	10	0	1.000	0.001	1.000	Excellent
12	10	0	1.000	0.001	1.000	Excellent
13	10	0	1.000	0.001	1.000	Excellent
14	10	0	1.000	0.001	1.000	Excellent
15	10	0	1.000	0.001	1.000	Excellent
16	10	0	1.000	0.001	1.000	Excellent
17	10	0	1.000	0.001	1.000	Excellent
18	10	0	1.000	0.001	1.000	Excellent

^a^Face validity index average for 18 items (understandability)=0.961.

^b^Computed using the formula: Pc = [(N!/A!) (N–A)!] * 0.5^N^, where Pc=probability of chance occurrence, N=number of experts, and A=number of experts who agree the items are relevant.

^c^Computed using the formula: κ = (item-level content validity index – Pc) / (1–Pc), where Pc=probability of chance occurrence. The interpretation criteria for kappa were as follows: Excellent=κ>0.7, Good=0.6≤κ≤0.74, and Fair=0.40≤κ≤0.59.

Of the 59 participants, 51 (the calculated sample size was 48) signed the web-based consent form and completed the questionnaire for the reliability study. The age of the participants ranged from 22 years to 25 years, and all of them were students (females, 47/51, 92%). The mean usability score for MyFitnessPal using M-MAUQ was 0.802 (SD 0.104), which indicated good usability. The Cronbach α values for total, subscale ease of use (Q1-Q5), subscale interface and satisfaction (Q6-Q12), and subscale usefulness (Q13-Q18) were .946, .893, .942, and .742, respectively. The value of Cronbach α if an item was deleted (refer to Table 4) remained highly consistent without significant difference, indicating good internal reliability of the developed questionnaire. The final M-MAUQ can be viewed in Multimedia Appendix 1.

**Table 4:** The internal consistency of the item total statistics.

Item	Scale mean if item deleted	Scale variance if item deleted	Corrected item total correlation	Cronbach α if item deleted
Q1	95.31	156.660	0.789	.941
Q2	95.12	161.866	0.761	.942
Q3	95.37	158.558	0.674	.943
Q4	95.39	160.323	0.706	.942
Q5	95.49	158.135	0.650	.944
Q6	95.41	158.487	0.795	.941
Q7	95.29	159.812	0.776	.941
Q8	95.06	160.856	0.794	.941
Q9	95.41	158.407	0.751	.941
Q10	95.31	156.900	0.825	.940
Q11	95.57	155.370	0.849	.939
Q12	95.27	159.723	0.868	.940
Q13	95.18	160.868	0.745	.942
Q14	95.10	162.570	0.759	.942
Q15	95.31	162.500	0.727	.942
Q16	95.47	161.454	0.655	.943
Q17	97.35	180.113	–0.057	.961
Q18	95.24	165.304	0.703	.943

## Discussion

This study described the translation, adaptation, and validation of the English version of MAUQ into the Malay version. The results of our study revealed a high level of content validity and face validity for M-MAUQ (standalone for patients). The content validity index was high for all individual items (item-level content validity index >0.900) and for overall score (scale content validity index >0.944) in term of relevancy and clarity, which exceeded the recommended benchmark of 0.79 [21]. Moreover, our kappa statistic indicated excellent agreement between expert raters for content validity and among user raters for face validity (κ>0.77). This study also had high reliability for the total domains of M-MAUQ, with Cronbach α>.90.

A questionnaire is one of the well-known methods for usability testing [27], but developing a new one might require concerted effort by the members of a research team, extra cost, and a lot of time [19]. Thus, adaptation of established, appropriate, and available questionnaires with documented validity in other languages is recommended [19]. Appropriate translation and adaptation of an instrument are essential for ensuring the equivalence between the original and translated versions translated according to the accepted standards and at the same time being culturally and conceptually appropriate [28]. Literal translation might cause misunderstanding of the questions posed since it does not anticipate the cultural sensitivity or the cultural influence of the question. Regardless of the presence of a multi-ethnic community in Malaysia, Bahasa Malaysia or Malay is the national language, which justifies validating a Malay-translated version for MAUQ [29]. In this study, we translated, culturally adapted, and validated an 18-item questionnaire called the M-MAUQ. This study adheres to the comprehensive and detailed guidelines for the translation, adaptation, and validation process documented by Sousa and Rojjanasrirat [20], which was adopted by Marzuki et al [19]. Translating some technical words such as navigation and interphase were challenging since these words were more familiar in English than in Malay. Moreover, a direct translation of the words of “social setting” from the questionnaire might raise questions from the user regarding the meaning. Thus, the involvement of certified translators who are experts in the translated and original language is essential. Harmonization among experts is essential to ensure that the user has understood the sentence correctly. A few examples were added to make the questions more clear and discussions were held with the original authors of MAUQ to ensure the consistency of each item in both languages.

Validation is an essential process for assuring that the measures in the translated version of the questionnaire are equivalent to those of the construct of the original version. Numerous studies have established content validity by using content validity index to quantify the validity of the questionnaire [27]. Content validity index is used primarily by researchers because it is simple to measure, is understandable, provides details for each item, and can be used to modify or delete instrument items [21]. Meanwhile, face validity focused more on the appearance and the understandability of the questionnaire by the target user. The validation process involved both experts and target users to ensure that different opinions from the 2 groups could be collected and discussed, and a consensus could be reached [21]. Modified kappa informed the agreement properties among the raters [30], and high scores in both content validity index and face validity index suggest that M-MAUQ had been translated accurately and adopted appropriately by our local users.

The internal consistency of the questionnaire is one of the reliability components. Internal consistency was determined by calculating the Cronbach α coefficient that represents the extent to which the items are measuring the same things [31,32]. The results of the reliability of M-MAUQ were comparable to those of the English MAUQ, which was tested among 128 participants in the Greater Pittsburgh area and the Cronbach α coefficients for the 3 subscales ranged from .717 to .908 [11].

This study utilized the web-based data collection method owing to the current COVID-19 pandemic, wherein mass gathering is not encouraged. Participants were gathered through virtual meetings using the Google meet platform. Although virtual meetings offer few advantages such as flexible time schedules and flexibility in attending the meeting from each one’s own comfort zone, some limitations were identified. Technical issues such as sudden internet breakdown or slowdown could have led to communication breakdown. However, participants who had problems with internet connection were contacted later in separate virtual meetings. The other limitations were as follows. First, the convenient sampling method was used, wherein participants were recruited from 1 university in the state of Pahang, Malaysia, thereby making it difficult to generalize the findings of this study to a wider population in Malaysia. Second, this research only focused on healthy young adults and the findings may not be generalizable to older adults or anyone with health-related issues. Third, additional reliability steps of test-retest would be beneficial for measuring the stability of respondent attributes.

In conclusion, our study shows that M-MAUQ has excellent validity and reliability. M-MAUQ can be used to assess the usability of mHealth apps among healthy young adults who are native Malay speakers. We suggest conducting a criterion validation study for future research [33,34] as this would be able to predict an outcome for another measure or domain of the M-MAUQ. Moreover, this questionnaire can be added in the future usability rating scale, as suggested in the Good Practice Guidelines on Health Apps and Smart Devices [35].

## Appendix

Multimedia Appendix 1 Actual Malay version of the mHealth App Usability Questionnaire (M-MAUQ).

## Abbreviations

MAUQ: mHealth app usability questionnaire
mHealth: mobile health
M-MAUQ: Malay version of the mHealth app usability questionnaire
PACMAD: People at the Centre of Mobile App Development
