# Supplementary material for: Malay Version of the mHealth App Usability Questionnaire (M-MAUQ): Translation, Adaptation, and Validation Study
Source: JMIR Mhealth Uhealth. 2021 Feb 4;9(2):e24457. doi: 10.2196/24457 (PMC7894394; doi:10.2196/24457)
Supplement: Multimedia Appendix 1 [file mhealth_v9i2e24457_app1.pdf]

Malay-mHealth Application Usability Questionnaire (M-MAUQ) Standalone

Sila tandakan jawapan yang paling sesuai berdasarkan skala seperti berikut:

1 Sangat tidak setuju

2 Tidak setuju

3 Agak tidak setuju

4 Neutral

5 Agak setuju

6 Setuju

7 Sangat setuju

	**1**	**2**	**3**	**4**	**5**	**6**	**7**
1. Aplikasi ini mudah digunakan							
2. Mudah untuk saya belajar cara menggunakan aplikasi ini							
3. Navigasi (petunjuk arah) antara paparan skrin adalah konsisten.							
4. Antara-muka aplikasi membolehkan saya menggunakan semua fungsi yang ditawarkan. (contoh: memasukkan maklumat, memberi respons terhadap peringatan, melihat maklumat)							
5. Apabila saya melakukan kesilapan semasa menggunakan aplikasi, proses pembetulan adalah mudah							
6. Saya suka antara-muka aplikasi ini							
7. Maklumat dalam aplikasi teratur, membantu saya mencari maklumat yang diperlukan dengan mudah							
8. Aplikasi ini menerima dan memberi maklum balas secukupnya tentang perkembangan aktiviti saya (contoh maklum balas: maklumat anda berjaya disimpan)							
9. Saya berasa selesa menggunakan aplikasi ini dimana-mana sahaja							

walaupun bersama dengan orang lain seperti rakan-rakan dan keluarga							
10. Masa yang diperlukan untuk menggunakan aplikasi ini sesuai dengan saya							
11. Saya akan menggunakan lagi aplikasi ini							
12. Secara keseluruhannya saya suka aplikasi ini							
13. Aplikasi ini bermanfaat untuk kesihatan dan kesejahteraan saya							
14. Aplikasi ini meningkatkan akses saya kepada perkhidmatan penjagaan kesihatan							
15. Aplikasi ini membantu menguruskan kesihatan saya dengan berkesan							
16. Aplikasi ini mempunyai semua fungsi dan kebolehan yang saya harapkan							
17. Saya masih boleh menggunakan aplikasi ini walaupun tanpa internet							
18. Aplikasi ini memberi ruang penjagaan kesihatan yang baik (contoh: akses kepada bahan pendidikan, memantau aktiviti dan menilai status kesihatan diri)							
